# Supplementary material for: Raman spectroscopy reveals phenotype switches in breast cancer metastasis
Source: Theranostics. 2022 Jul 11;12(12):5351–63. doi: 10.7150/thno.74002 (PMC9330538; doi:10.7150/thno.74002)
Supplement: Supplementary file 1 — Supplementary figure and tables. [file thnov12p5351s1.pdf]

Supporting Information

Raman spectroscopy reveals phenotype switches in breast cancer metastasis

Santosh Kumar Paidi,[1,#,&] Joel Rodriguez Troncoso,[2,#] Mason G. Harper,[3] Zhenhui Liu,[1] Khue G. Nguyen,[4] Sruthi Ravindranathan,[5] Lisa Rebello,[6] David E. Lee,[7,$] Jesse D. Ivers,[2] David A. Zaharoff,[4] Narasimhan Rajaram,[2,8,*] Ishan Barman[1,9,10,*]

Affiliations:

[1]Department of Mechanical Engineering, Johns Hopkins University, Baltimore, MD, 21218

[2]Department of Biomedical Engineering, University of Arkansas, Fayetteville, AR 72701

[3]University of Arkansas for Medical Sciences, Little Rock, AR, 72205

[4]Joint Department of Biomedical Engineering, University of North Carolina and North Carolina State University, Raleigh, NC, 27695

[5]Department of Hematology and Oncology, Emory University, Atlanta, GA, 30322

[6]Cell and Molecular Biology Program, University of Arkansas, Fayetteville, AR 72701

[7]Department of Health, Human Performance, and Recreation, University of Arkansas, Fayetteville, AR, 72701

[8]Winthrop P. Rockefeller Cancer Institute, University of Arkansas for Medical Sciences, Little Rock, AR, 72205

[9]The Russell H. Morgan Department of Radiology and Radiological Science, The Johns Hopkins University School of Medicine, Baltimore, MD, 21205

[10]Department of Oncology, Johns Hopkins University, Baltimore, MD, 21287

[#]Both authors contributed equally to this work.

[&] Current affiliation – School of Optometry, University of California, Berkeley, CA, 94720

[$] Current affiliation – Duke Molecular Physiological Institute, Duke University, 300 N. Duke St., Durham, NC 27701

*Corresponding authors: Ishan Barman (ibarman@jhu.edu) and Narasimhan Rajaram (nrajaram@uark.edu)

The authors disclose no potential conflicts of interest.

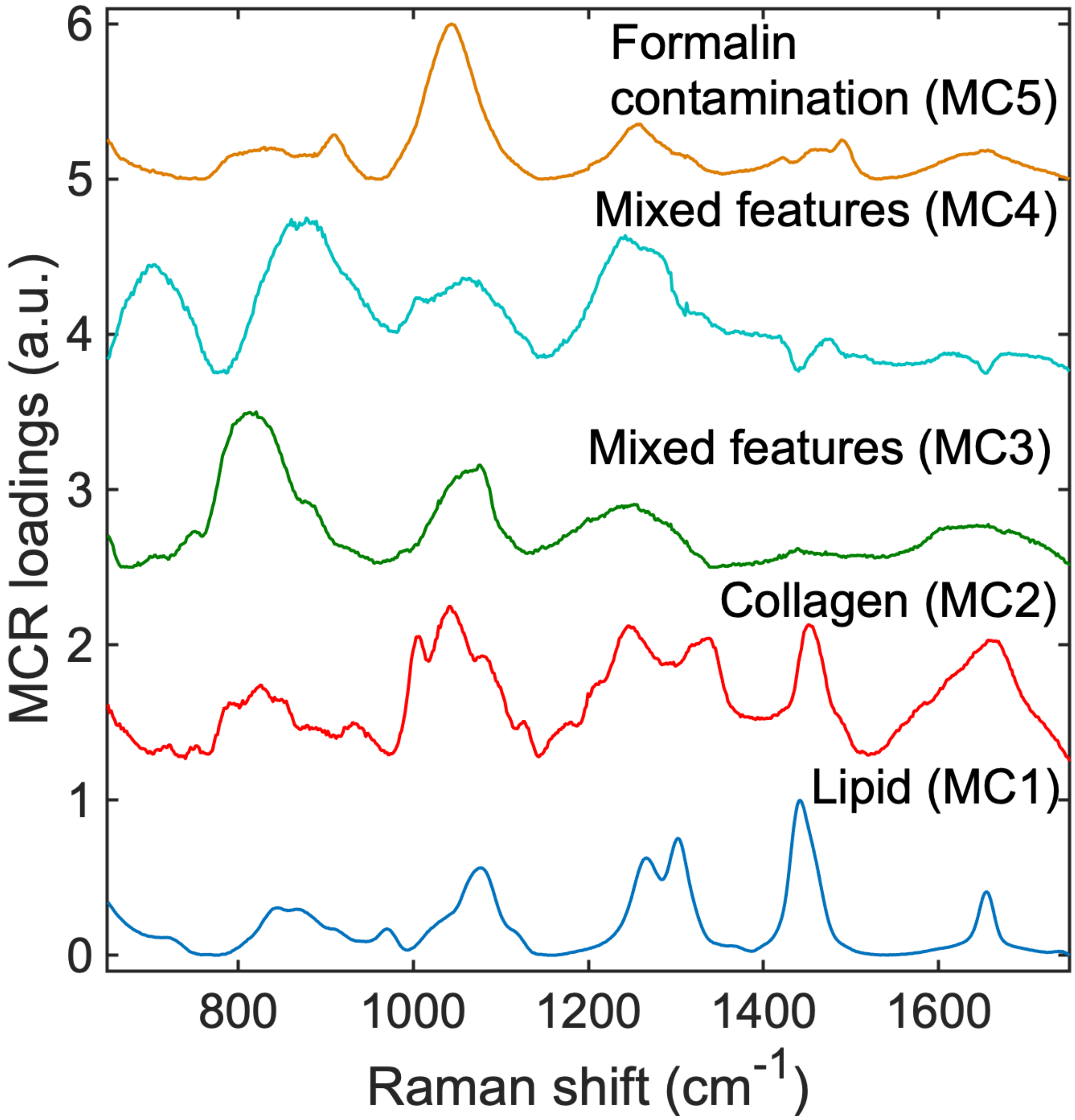

Figure S1. The complete set of MCR-ALS component loadings are provided for the entire Raman spectral dataset.

Table S1. Table of MCR-ALS component spectral peak assignments

Observed Raman peaks in the MCR loadings (cm^{-1})					Raman band assignment from literature
**MC1**	**MC2**	**MC3**	**MC4**	**MC5**	
			703		Cholesterol
		813			O-P-O stretching in DNA and RNA; quartz contribution
	851				C-C stretch of proline in collagen
			878		C-C stretching, hydroxyproline of collagen
				910	Formalin contamination during tissue fixation
	931				C-C vibration in collagen backbone
	1003		1003		Phenylalanine of collagen
	1042				Proline in collagen
				1044	Formalin contamination during tissue fixation
			1063		Skeletal C-C stretch of lipids
		1076			PO_2^- symmetric stretching in DNA
1078					C-C stretch
	1082				Carbohydrate residues of collagen
		1239	1242		PO_2^- asymmetric stretching in DNA
	1251				Amide III in collagen
				1256	Formalin contamination during tissue fixation
1266					CH_2 in-plane deformation (Triglyceride)
1302					CH vibration (Triglyceride)
	1337				CH_3CH_2 wagging modes of collagen
1442					CH_2 bending mode (Triglyceride)
	1451				CH_2 bending mode in collagen
				1491	Formalin contamination during tissue fixation
1654					C=C lipid stretch
	1657				α-helical structure of amide I in collagen

Table S2. Assignment for the top spectral predictors derived from random forest analysis

Observed Raman peaks	Raman band assignment from literature
670	C-S stretching mode of cystine (collagen type I)
862	Tyrosine (collagen)
962	Symmetric stretching vibration of PO_4^{3-}
1042	Proline (collagen)
1098	C-N stretch (lipid)
1213	C-N stretch
1254	Amide III
1330	Collagen
1372	Ring breathing modes of DNA/RNA bases
1432	CH_2 deformation (lipid)
1462	CH_2/CH_3 deformation (lipid and collagen)
1566	Tryptophan
1664	Amide I (collagen)
